# Supplementary material for: From growers to patients: Multi-stakeholder views on the use of, and access to medicinal cannabis in Australia
Source: PLoS One. 2022 Nov 11;17(11):e0277355. doi: 10.1371/journal.pone.0277355 (PMC9651563; doi:10.1371/journal.pone.0277355)
Supplement: S1 File — (DOCX) [file pone.0277355.s001.docx]

**S1 File – list of stakeholders included in the analysis, n = 121**

Link to parliamentary inquiry: <https://www.aph.gov.au/Parliamentary_Business/Committees/Senate/Community_Affairs/Medicinalcannabis>

| **Stakeholder group** | **Included submissions** |
| --- | --- |
| **Governmental organisations (n=5)** | Northern Territory Government |
|  | Department of Health |
|  | Queensland Government |
|  | Department of Veterans' Affairs |
|  | Northern Territory government |
| **Non-governmental organisations** | |
| **Professional health bodies (n=7)** | Society of Hospital Pharmacists of Australia |
|  | Royal Australian College of General Practitioners |
|  | Pharmaceutical Society of Australia |
|  | Royal Australian and New Zealand College of Psychiatrists |
|  | Australian Medical Association |
|  | Australian and New Zealand Society of Palliative Medicine |
|  | Clinical Oncology Society of Australia |
| **Health charities, patient and consumer advocacy groups (n=18)** | Alcohol and Drug Foundation |
|  | Medicinal Cannabis Industry Australia |
|  | United in Compassion |
|  | Medical Cannabis Users Association of Australia |
|  | Medical Cannabis Knowledge Network |
|  | Epilepsy Action Australia |
|  | Cancer Voices Australia |
|  | Medical Cannabis Council |
|  | Pharmacy Guild of Australia |
|  | Medical Cannabis Users Assocation of Tasmania |
|  | Country Women’s Association of Australia |
|  | Drug Free Queensland |
|  | Drug Free Australia |
|  | New South Wales Nurses and Midwives’ Association |
|  | Multiple Sclerosis Research Australia and Multiple Sclerosis Australia |
|  | Australasian College of Nutritional and Environmental Medicine |
|  | Queensland Nurses and Midwives' Union |
|  | Australian Pain Management Association |
| **Pharmaceutical and medicinal cannabis industry (n=16)** | LeafCann Group |
|  | Entoura Pty Ltd |
|  | Nimbin HEMP Embassy |
|  | Cann Group Limited |
|  | Canopy Growth Australia |
|  | CANNATREK LTD |
|  | Ecofibre |
|  | Little Green Pharma |
|  | Tilray |
|  | GW Pharmaceuticals |
|  | Tasmanian Alkaloids |
|  | FreshLeaf Analytics |
|  | MedReleaf Australia |
|  | Parsl |
|  | AusCann Group Holdings Ltd |
|  | Bod Australia |
| **Member of the public (n=77)** | |
| **Patients and caregivers (n=63)** | Anonymous (names Withheld) |
| **Health professionals, academics, or research centres (n=12)** | Monday Discussion Group of Residents of St Vincent’s Kangaroo Point |
|  | Associate Professor Kate Seear and Springvale Monash Legal Service |
|  | NICM Health Research Institute (Western Sydney University) |
|  | Australian Centre for Cannabinoid Clinical and Research Excellence |
|  | Applied Cannabis Research |
|  | Lambert Initiative |
|  | Professor James Angus |
|  | Professors Wayne Hall and Michael Farrell |
|  | Dr Helen Jarvis |
|  | Professor Laurence Mather |
|  | CA Clinics |
|  | Mr Simon Eckermann |
